# Evidence for Distinct Forms of Compulsivity in the SAPAP3 Mutant-Mouse Model for Obsessive-Compulsive Disorder

**DOI:** 10.1523/ENEURO.0245-19.2020

**Published:** 2020-04-21

**Authors:** I. Ehmer, L. Crown, W. van Leeuwen, M. Feenstra, I. Willuhn, D. Denys

**Affiliations:** 1 Netherlands Institute for Neuroscience, Royal Netherlands Academy of Arts and Sciences, 1105 BA Amsterdam, The Netherlands; 2 Department of Psychiatry, Amsterdam UMC, University of Amsterdam, 1105 AZ Amsterdam, The Netherlands

**Keywords:** compulsivity, feedback processing, obsessive-compulsive disorder, SAPAP3 knock-out mice, signal attenuation

## Abstract

The specific mechanisms underlying compulsive behavior in obsessive-compulsive disorder (OCD) are unknown. It has been suggested that such compulsivity may have its origin in cognitive dysfunction such as impaired processing of feedback information, received after the completion of goal-directed actions. The signal attenuation (SA) task models such a processing deficit in animals by attenuating the association strength between food reward and audiovisual feedback (signal) presented after performance of an operant response. The compulsive-like responding resulting from SA is well characterized in rats, but was so far not established in mice, a species for which powerful genetic OCD models exist. Thus, first, we demonstrate that the SA task can be implemented in mice and show that attenuation of reward-associated response feedback produces similar behavior in C57BL/6 mice as previously reported in rats. Second, we tested the hypothesis that *SAPAP3* knock-out mice (SAPAP3^-/-^), prone to exhibit several OCD-like abnormalities including excessive grooming, show enhanced compulsive-like behavior in the SA task compared with their wild-type (WT) littermates. However, task-related compulsivity measures in SAPAP3^-/-^ and WT did not yield significant differences, neither following SA nor during “regular” extinction of operant behavior. Thus, compulsive-like instrumental behavior following feedback distortion was not potentiated in compulsively grooming mice, implicating specifically that (1) a general deficit in feedback processing is not related to excessive grooming in SAPAP3^-/-^ and (2) different manifestations of compulsivity may be driven by independent mechanisms.

## Significance Statement

The signal attenuation (SA) task is a well-established behavioral paradigm for rats that promotes compulsivity. First, we demonstrate that the SA task can also be applied to test feedback processing in mice. Second, we investigated whether SAPAP3 mutant mice, a highly validated genetic animal model for obsessive-compulsive disorder (OCD), exhibit exacerbated compulsive responding in the SA task. However, we found no enhancement of feedback-induced compulsivity in SAPAP3 mutants. Thus, our results indicate the existence of different types of compulsivity (i.e., behaviorally vs genetically induced compulsivity) that are likely driven by independent mechanisms.

## Introduction

Compulsive behavior is driven by the urge to perform repetitive actions in a rigid or stereotyped manner and by the experience of limited voluntary control over such an urge, including a diminished ability to delay or inhibit these behaviors (Denys, 2011). Compulsive behavior can be observed in a number of neurodegenerative and psychiatric disorders (Berlin and Hollander, 2014). In obsessive-compulsive disorder (OCD), patients have recurring, unwanted thoughts (obsessions) that make them feel driven to act (compulsions), often with the intention to prevent dreaded events or situations (Luigjes et al., 2019), despite insight into how unreasonable and inappropriate this behavior is (American Psychiatric Association, 2013). The specific processes underlying compulsive behavior are still unknown, but various hypotheses about impairment of cognitive functions have been put forward, such as a deficiency of feedback processing (Otto, 1992; Nielen et al., 2009), cognitive inflexibility (Chamberlain et al., 2006), reduced behavioral inhibition (Chamberlain et al., 2005; Morein-Zamir et al., 2010), imbalance between goal-directed and habitual behavior (Gillan et al., 2011), emotional dysregulation (Steketee et al., 1996; Szechtman and Woody, 2004; Endrass et al., 2011; Kaczkurkin and Lissek, 2013), or intolerance to uncertainty (Reuther et al., 2013). Animal models for OCD offer the possibility to study these cognitive impairments separately (Albelda and Joel, 2012; Camilla d'Angelo et al., 2014; Szechtman et al., 2017).

In the present study, we investigated whether impaired processing of external feedback underlies compulsive behavior in a genetic mouse model for OCD, the *SAPAP3* knock-out mouse (SAPAP3^-/-^). SAPAP3^-/-^ self-groom excessively and display increased anxiety and decreased behavioral flexibility (Welch et al., 2007; Manning et al., 2019; van den Boom et al., 2019). Compulsive-like grooming aggravates during aging and may continue to the point that the animals develop grooming-induced facial hair loss and skin lesions. This excessive self-grooming bears similarity to symptoms such as compulsive hand-washing observed in OCD patients, hair-pulling in trichotillomania patients, or nail-biting in onychophagia patients (Welch et al., 2007; Yang and Lu, 2011). Similar to OCD patients, compulsive grooming can be normalized by administration of selective serotonin reuptake inhibitors (SSRIs) or deep-brain stimulation (Welch et al., 2007; Pinhal et al., 2018).

Impaired feedback processing has been modeled in the signal attenuation (SA) task, developed for rats by Joel and colleagues (Joel and Avisar, 2001; Joel, 2006; Albelda and Joel, 2012). This task is based on the assumption that compulsive behavior can be caused by deficient processing of environmental cues that signal the completion of goal-directed behavior. In this sense, such external response feedback resembles characteristics of perceptual signals, but not internal reference or error signals described in cybernetic models (Pitman, 1987). In the SA task, animals learn that an operant response leads to the delivery of food pellets and that an audiovisual signal provides response feedback. To simulate feedback deficiency experimentally, the incentive salience of this signal is attenuated by presenting it in absence of food delivery. This leads to compulsive-like responding (in a subsequent extinction test) that is absent in animals not exposed to this SA treatment and may resemble repetitive, inappropriate, and compulsive behavior that OCD patients are unable to suppress (Joel, 2006). This notion is supported by a decrease in compulsive responding in SA-exposed animals following interventions with treatments effective in OCD (Joel, 2006).

Similar brain circuits are thought to underlie compulsive states induced by SA and by genetic deletion of the SAPAP3 protein. Inactivation of the lateral orbitofrontal cortex (lOFC) potentiated (Joel et al., 2005a,b) or induced (Joel and Klavir, 2006) compulsive lever-pressing in an SA task, whereas SAPAP3^-/-^ show abnormalities in lOFC neuronal activity and perturbed cortico-striatal network activation (Lei et al., 2019). Moreover, stimulation of the lOFC-striatal pathway alleviates excessive grooming (Burguière et al., 2013). Notably, such dysfunction seems to be restricted to cortico-striatal circuits, and not extend to thalamo-cortical circuits (Wan et al., 2014). However, the involvement of cortico-striatal pathways other than projections from the lOFC need further investigation. For example, striatal input from the secondary motor cortex, which is strengthened in SAPAP3^-/-^ (Corbit et al., 2019), has not yet been tested in SA.

Under the assumptions that a general deficit in feedback processing is a major source of compulsive behavior (via a shared underlying neuronal pathology) and that compulsivity is a unitary and uniform phenomenon, then compulsivity in the SA task should be exacerbated in animal models for OCD (which already display compulsivity before SA induction of compulsivity). Therefore, we subjected SAPAP3^-/-^ (genetic OCD model) to the SA task, hypothesizing that SAPAP3^-/-^ with SA-induced feedback deficiency would show more compulsive responding than normal wild-type (WT) control mice, comparable to the finding of Sesia et al. (2013) that revealed enhanced compulsivity in the SA task after repeated quinpirole administration (pharmacological OCD model). Alternatively, a variety of neural mechanisms might independently cause qualitatively different forms of compulsivity that do not potentiate each other. In this case, SA-induced compulsivity would not differ between SAPAP3^-/-^ and WT. To test these hypotheses, we first implemented and validated the SA task, previously exclusively used in rats, in C57BL/6 mice (experiment 1). In a second step, we trained SAPAP3^-/-^ in this task and compared their behavior to that of WT mice (experiment 2). Furthermore, self-grooming of SAPAP3^-/-^ was scored in different environmental contexts.

## Methods and Materials

### Subjects

To validate the SA task in mice, 24 C57BL/6JRccHsd, male mice were obtained from Harlan (experiment 1). To test the hypothesis that impaired feedback would underlie compulsive behavior, SAPAP3^-/-^ (bred on a C57BL/6J background; founders provided by Dr. Guoping Feng, Massachusetts Institute of Technology) and their WT littermates were bred in house. 34 SAPAP3^-/-^ (17 male, 17 female) and 35 WT (18 male, 17 female) were included in this study (experiment 2). At the start of behavioral training, all animals were three to four months of age, individually housed in an environment with reversed day-night cycle (12/12 h dark/light), controlled temperature, and humidity. Training and testing were performed in the animals’ active period. All mice were food-restricted to a target weight of 90% of their individual ad-libitum weight, while water intake was *ad libitum*. Weight and health of the animals were monitored on a daily basis and special attention was paid to the formation of lesions in SAPAP3^-/-^. Before experimental training, all mice were handled for three consecutive days (Hurst and West, 2010). Apparatus, procedure, and statistical analysis were identical for experiments 1 and 2. All animal procedures were performed in accordance with the Dutch law and the Royal Netherlands Academy of Arts and Sciences animal care committee’s regulations.

### Apparatus

Behavioral training and testing was conducted in standard operant boxes (Med-Associates), housed within sound-attenuated chambers. Each box was equipped with a food magazine on one wall and a house light (3 W, 24 V) on the opposite side. In some sessions, nose-poke holes and signaling lights were installed next to the food magazine. Food magazine and nose-poke holes contained infra-red beams for the detection of the animals’ responses. The food magazine could be illuminated by a 3-W light and was connected to a pump with a syringe that delivered bouts of 20 μl of 20% sucrose solution. In addition, a speaker was attached to each chamber that produced tones with 80 dB and 2.8 kHz. Animal behavior was videotaped. All task programming and data acquisition was performed with Med-PC-IV software (Med-Associates).

### Procedure

**Figure 1.**
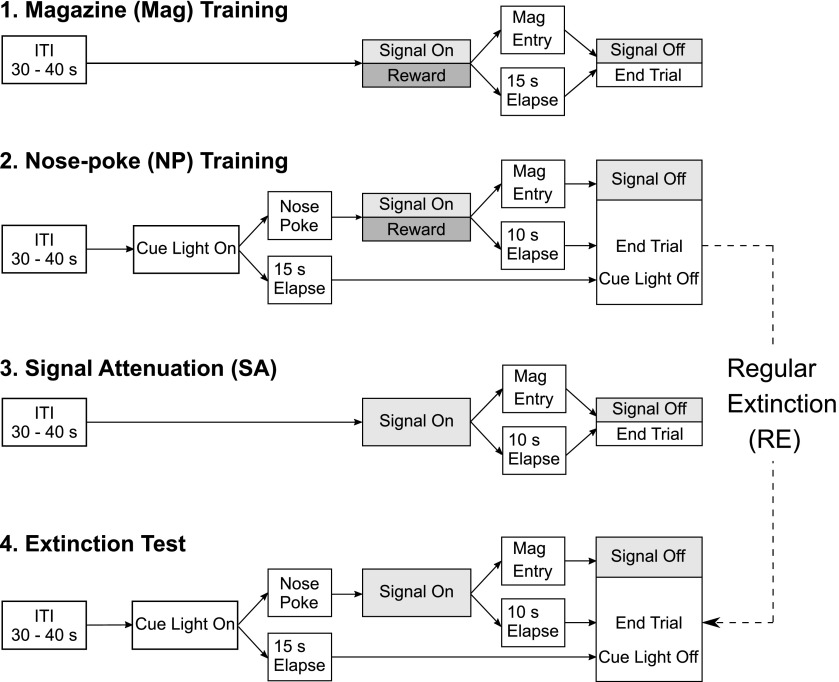
Experimental design of the mouse SA task. Training and testing procedures are based on the original rat version of the SA task (Joel and Avisar, 2001; Joel, 2006). At the beginning of the task animals learn to associate delivery of a food reward with an audiovisual feedback signal that indicates that food is available in the food magazine (stage 1, Mag training). Thereafter, animals learn that making a nose poke during illumination of a cue light leads to delivery of a food reward, accompanied by the signal (stage 2, nose-poke training; NP). Subsequently, half of the animals are exposed to the SA procedure (stage 3, SA) in which the information value of the signal is decreased by presenting the signal without food reward (simulation of feedback deficiency). Finally, all animals are tested under extinction conditions (stage 4, extinction test). Compulsivity measures (UCTs, ENPs) will be compared between animals that were exposed to SA prior and those that were not (experienced the extinction test only, i.e., RE without prior manipulation of the feedback signal).

The experimental design of the SA task consisted of four consecutive stages and was based on the rat SA task (Joel and Avisar, 2001; Joel, 2006; Fig. 1).

#### Stage 1: shaping and magazine (Mag) training

During the initial two shaping sessions, animals were placed in the operant chamber for 30 min to adapt to the new environment and to find the site of reinforcement (food magazine). The house light and the magazine light were illuminated and the food magazine was filled with sucrose solution. In the subsequent Mag training, mice learned to associate a compound signal (tone and magazine light) with delivery of the sucrose reward into the food magazine (i.e., feedback signal). Each trial started with an intertrial interval (ITI) of 30–40 s, followed by delivery of the reward and simultaneous presentation of the feedback signal. A trial ended after the animal entered the food magazine (after signal “on”) or 15 s elapsed. Both conditions caused the feedback signal to turn off. Each mouse was required to collect 20 out of 30 possible rewards in two successive sessions to proceed to training stage 2. During shaping and Mag training, nose-poke holes were not installed.

#### Stage 2: nose-poke training

In this training stage, mice learned to make a nose-poke response into a hole placed to the left or right of the food magazine. One poke hole was designated the correct hole and poking into this hole initiated reward delivery, whereas a response in the other hole was never rewarded. The side of correct and incorrect holes was counterbalanced across animals. Each training trial started with an ITI of 30–40 s. Thereafter, a cue light indicated that a response was required. When the animal poked in the correct hole, a reward was delivered in the food magazine signaled by simultaneous presentation of the feedback signal. Thus, the compound signal provided feedback about the completed action, the nose poke caused a reward delivery into the food magazine. When the animal collected the reward within the 10 s of the start of feedback signal presentation, this was recorded as a completed trial (CT) and the feedback signal turned off. A failure to collect the reward during the feedback signal presentation was recorded as an uncompleted trial (UCT). In this case, the cue light and the feedback signal turned off after 10 s. No-poke trials were trials in which the animal did not respond in the correct poke hole, regardless of responses in the incorrect poke hole. These trials were terminated after 15 s without presentation of the feedback signal. Nose-poke responses that were performed in addition to the required initial poke, were recorded as extra nose-pokes (ENPs) and were never rewarded. Training was conducted in two training stages. First, animals were required to collect at least 24 out of 30 possible rewards in CTs in two successive training sessions. Thereafter, the final nose-poke training stage, consisting of 50 trials, required successful completion of 34 CT trials.

#### Stage 3: signal attenuation (SA)

After nose-poke training, all animals were randomly assigned to either the SA or the regular extinction (RE) condition. For SA, the nose-poke holes were covered with metal plates and the feedback signal was presented in 30 trials without being paired with reward. The number of entries into the food magazine was recorded to provide information about the attenuation process. Each animal in the SA condition received three sessions of SA, with a maximum of two sessions per day. Animals assigned to the RE condition continued from nose-poke training (stage 2) directly to the final extinction test (stage 4), without any additional training.

#### Stage 4: extinction test

The final test consisted of a single session of 50 trials. For this test, the nose-poke holes were installed, but responding only led to presentation of the feedback signal of maximal 10 s but not to delivery of the sucrose reward (extinction conditions). In this extinction test, CTs, UCTs, and ENPs in completed (ENP in CT) and uncompleted (ENP in UCT) trials were measured.

### Grooming and anxiety

Grooming was measured for SAPAP3^-/-^ and WT. Behavior was recorded for 1 h in an open field (OF), a Plexiglas box (30 × 30 × 40 cm). An automated procedure of behavioral scoring was used to identify episodes of self-grooming and to determine locomotion (Van den Boom et al., 2017). A trained experimenter, who was blinded for genetic background of the animals, manually analyzed levels of self-grooming during the final extinction test. Anxiety-like behavior was assessed before the SA task on an elevated plus maze (EPM; 53 cm above the floor) with two closed (walls: width 4.5 cm, length 30 cm, height 15 cm) and two open arms (same dimensions but without walls). Animals were placed at the center of the maze. Time spent in open, closed, and center areas over a period of 5 min, as well as frequency of entries into these areas, were analyzed.

### Data processing

Data acquisition was performed with Med-PC-IV and preprocessed with MATLAB. Statistical analysis was conducted with IBM SPSS software. In-depth data analysis was performed for the last session of nose-poke training and the final extinction test. Dependent variables were the numbers of trials, specified as CTs, UCTs, and no-poke trials, as well as the numbers of nose-pokes, specified as the number of ENPs in CTs (ENP-CT) and in UCTs (ENP-UCT). Compulsive nose-poking was operationally defined as the number of ENP-UCTs (Joel, 2006). As data generally deviated from the assumption of normality, for all analyses non-parametric testing was used. Repeated measures analysis was performed with a Friedman test, followed by Wilcoxon signed-rank tests. Kruskal–Wallis *H* tests provided the χ^2^ statistic for analyzing non-parametric data with more than two independent samples, while Mann–Whitney *U* tests were employed for comparison of two independent samples. In case data were normally distributed repeated-measures ANOVA was employed. The threshold for statistical significance was set at *p* < 0.05. Results are presented as mean ± SEM. To compare compulsivity of SAPAP3^-/-^ and WT, genotypes were matched on task performance, resulting in exclusion of three SAPAP3^-/-^ (two SA, one RE) and five WT (one SA, four RE) from the final analysis due to decreased learning performance or equipment failure. Therefore, we report data of 31 SAPAP3^-/-^ and 30 WT.

## Results

### C57BL/6 mice readily acquired the response requirements in the SA task

**Figure 2.**
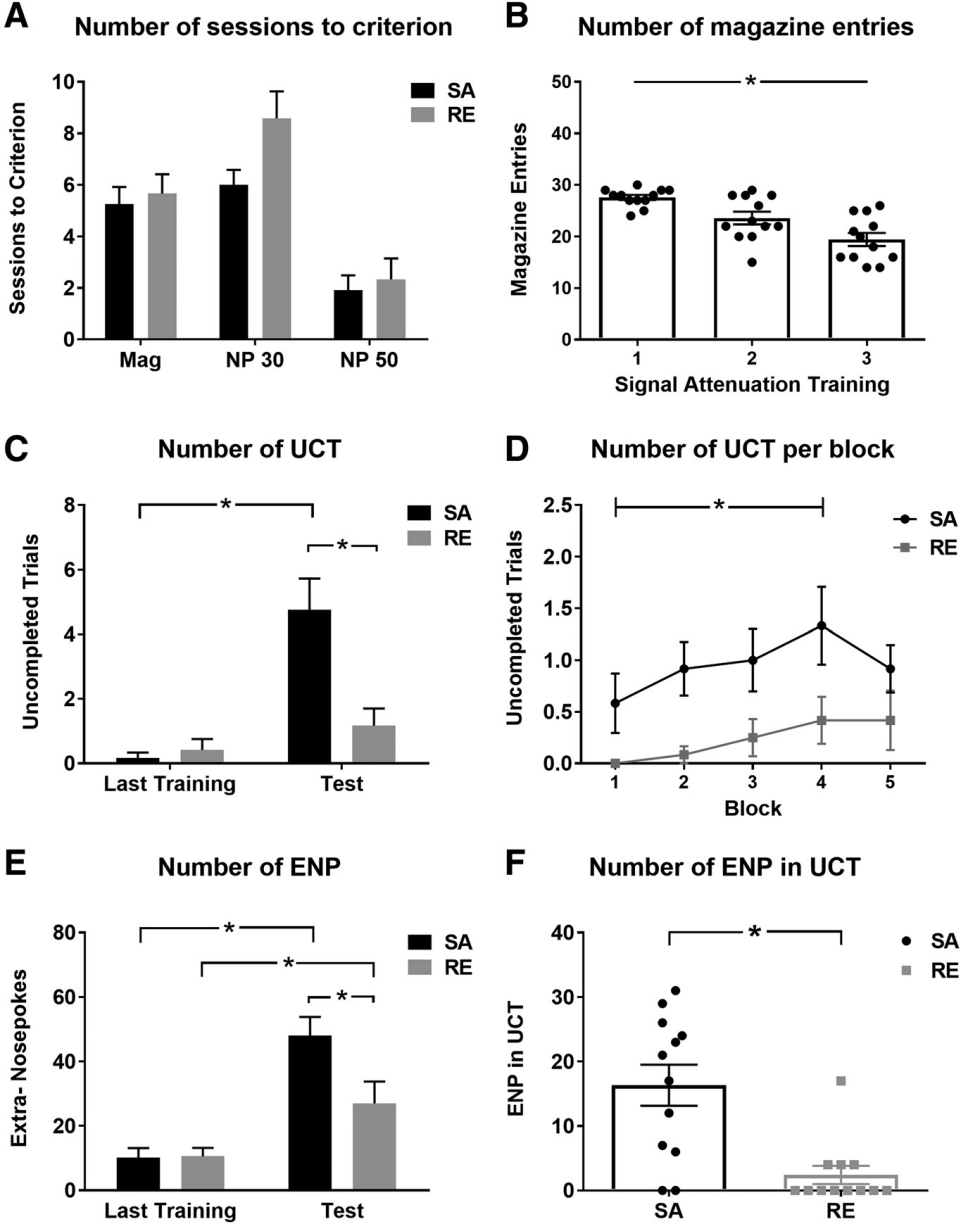
Validation of the SA task in mice. ***A***, Number of training sessions until reaching criteria (in Mag and nose-poke training; stages 1 and 2). Stage 2 consisted of two parts: nose-poke training with 30 trials (NP 30) and nose-poke training with 50 trials (NP 50). ***B***, Magazine entries of C57BL/6-SA mice declined across SA sessions (stage 3), demonstrating effective ‘attenuation’ of the feedback signal (i.e., attenuation of the association strength between signal and reward). ***C***, In the final extinction test (stage 4), C57BL/6-SA mice (*n* = 12) showed significantly more UCTs in comparison with C57BL/6-RE mice (i.e., mice that underwent only RE in stage 4 without prior SA; *n* = 12). ***D***, The mean number of UCT was significantly higher in SA mice in four out of five trial blocks. ***E***, All animals increased the number of ENPs in the final extinction test. However, ENP of C57BL/6-SA mice were significantly higher compared with C57BL/6-RE mice in the final extinction test (stage 4). ***F***, Examination of ENP in UCT showed increased compulsivity in C57BL/6-SA compared with C57BL/6-RE, demonstrating successful implementation of the SA task in mice. Mag, food magazine; NP, nose poke. Data are expressed as mean ± SEM. *p < 0.05.

All C57BL/6 acquired the SA task and completed the final extinction test (Fig. 2*A*). During the initial training stages C57BL/6 required 2–10 sessions of Mag training (5.3 ± 0.489), 4–15 training sessions in the first step of nose-poke training (7.3 ± 0.644), and accomplished the final nose-poke training in 1–10 sessions (2.1 ± 0.486). A Kruskal–Wallis test verified that there was no statistically significant difference in the number of training sessions between SA (*N* = 12) and RE (*N* = 12) condition (Mag, χ^2^(1) = 0.17, *p *=* *0.60; nose-poke 30, χ^2^(1) = 3.07, *p* = 0.08; nose-poke 50, χ^2^(1) = 0.04, *p* = 0.84). A Friedman test confirmed that the SA procedure was effective in attenuating the signal as marked by a significant reduction of entries into the food magazine following presentation of the signal (χ^2^(2) = 16.04, *p* < 0.001; Fig. 2*B*).

### Compulsive-like behavior is increased in C57BL/6 in the signal-attenuation condition

Our results show a number of significant differences between animals that underwent SA and animals that experienced a RE in the final test. First, the SA procedure provoked a significantly higher number of UCT in C57BL/6-SA than in C57BL/6-RE mice (*U* = 26.0, *p* = 0.006; C57BL/6-SA: 4.7 ± 0.97, C57BL/6-RE: 1.2 ± 0.53; Fig. 2*C*). Further analysis revealed a significant increase of UCT in C57BL/6-SA mice from the last nose-poke training [to the final extinction test (*Z* = −2.810, *p* = 0.005: last: 0.17 ± 0.17; test: 4.7 ± 0.97)] that was not observed in C57BL/6-RE mice (*Z* = −1.473, *p* = 0.141).

To investigate the within-session distribution of UCT we analyzed data of the final extinction test in blocks of 10 trials. Our results suggest that the SA produces consistent display of UCT as compared with RE (Fig. 2*D*). The number of CT was significantly higher in C57BL/6-RE mice (χ^2^(1) = 17.35, *p* < 0.001), whereas C57BL/6-SA mice had more no-poke trials (χ^2^(1) = 17.44, *p* < 0.001). Additionally, we recorded the number of ENPs. Similar to prior studies with rats, our results with mice confirmed that the SA procedure significantly increases the number of ENP (*U* = 28.5, *p* = 0.012; C57BL/6-SA: 46.1 ± 5.6, C57BL/6-RE: 25.0 ± 6.6; Fig. 2*E*). One of the most important markers of compulsive responding is the number of ENP in UCT. Our results reveal that the number of ENP in UCT was significantly higher in C57BL/6-SA compared with C57BL/6-RE (*U* = 19.0, *p* = 0.001; C57BL/6-SA: 16.2 ± 3.2, C57BL/6-RE: 2.3 ± 1.3; Fig. 2*F*). From these results we conclude that we successfully implemented the SA task for mice.

### Attenuation of the feedback signal in SAPAP3^-/-^ and WT was similar

**Figure 3.**
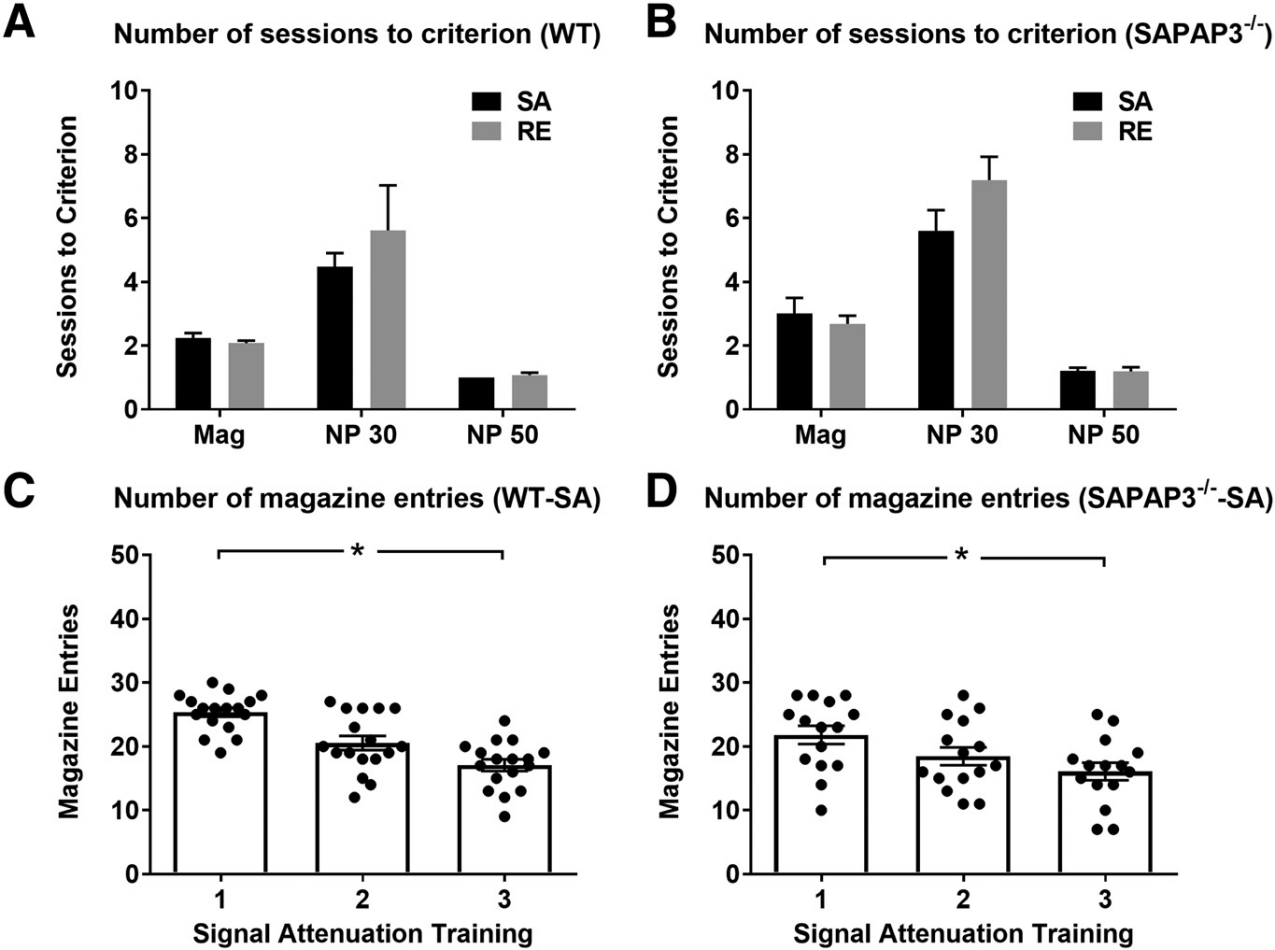
Training performance of SAPAP3^-/-^ and WT in the SA task. ***A***, Number of training sessions until reaching criteria (in Mag and nose-poke training; stages 1 and 2). Stage 2 consisted of two parts: nose-poke training with 30 trials (NP 30) and nose-poke training with 50 trials (NP 50). WT-RE and WT-SA required a similar number of training sessions to reach task criteria. ***B***, There was no difference between SAPAP3^-/-^-RE and SAPAP3^-/-^-SA in the number of training sessions until reaching task criteria. While SAPAP3^-/-^ required a few more sessions of Mag and NP 30 training than WT, overall genotype comparison in SA and RE condition did not reveal significant differences, confirming that both genotypes acquired the task equally. ***C***, Following NP 50 training, half of the WT and half of the SAPAP3^-/-^ were subjected to the SA procedure (stage 3, SA) in which the information value of the signal is decreased by presenting the signal without food reward (simulation of feedback deficiency). In WT-SA, magazine entries declined across three successive SA sessions, demonstrating effective attenuation of the feedback signal. ***D***, In SAPAP3^-/-^-SA, effective attenuation of the feedback signal was also demonstrated by decreasing magazine entries of across SA training sessions. No difference was observed between the number of magazine entries of WT and SAPAP3^-/-^, suggesting that the association between signal and reward was attenuated effectively in both genotypes (SAPAP3^-/-^: SA = 15, RE = 16; WT: SA = 17, RE = 13). Data are expressed as mean ± SEM. *p < 0.05.

Genotypes were matched on their performance during nose-poke training (stage 2), as we aimed to assess genotype differences during the extinction test only (stage 4). After nose-poke training, all mice were assigned to either the SA or the RE condition (within a genotype). Analyses confirmed that learning was not different between both conditions after this random assignment. WT-RE and WT-SA required a similar number of training sessions to reach task criteria in all stages of behavioral training (Fig. 3*A*). There was no difference between SAPAP3^-/-^-RE and SAPAP3^-/-^-SA in the number of training sessions until reaching task criteria (Fig. 3*B*).

The following SA procedure (stage 3, simulation of feedback deficiency) was analyzed with a two-factor ANOVA (session and genotype). Results show a significant effect of session (*F*_(2,60)_ = 32.267, *p* < 0.001), but no session-genotype interaction or genotype effect. ANOVAs per genotype confirmed that WT-SA magazine entries declined across three successive SA sessions (*F*_(2,32)_ = 38.6, *p* < 0.001; first session SA 25.8 ± 1.3; second session 20.5 ± 1.1; third session 17.0 ± 0.9), suggesting effective attenuation of the feedback signal (Fig. 3*C*). In SAPAP3^-/-^-SA, effective attenuation of the feedback signal was also demonstrated by decreasing magazine entries across SA training sessions (*F*_(2,28)_ = 7.09, *p* = 0.003; first session SA 21.8 ± 1.4; second session 18.5 ± 1.4; third session 16.1 ± 1.4; Fig. 3*D*).

### Compulsive-like responding in WT-SA versus WT-RE

**Figure 4.**
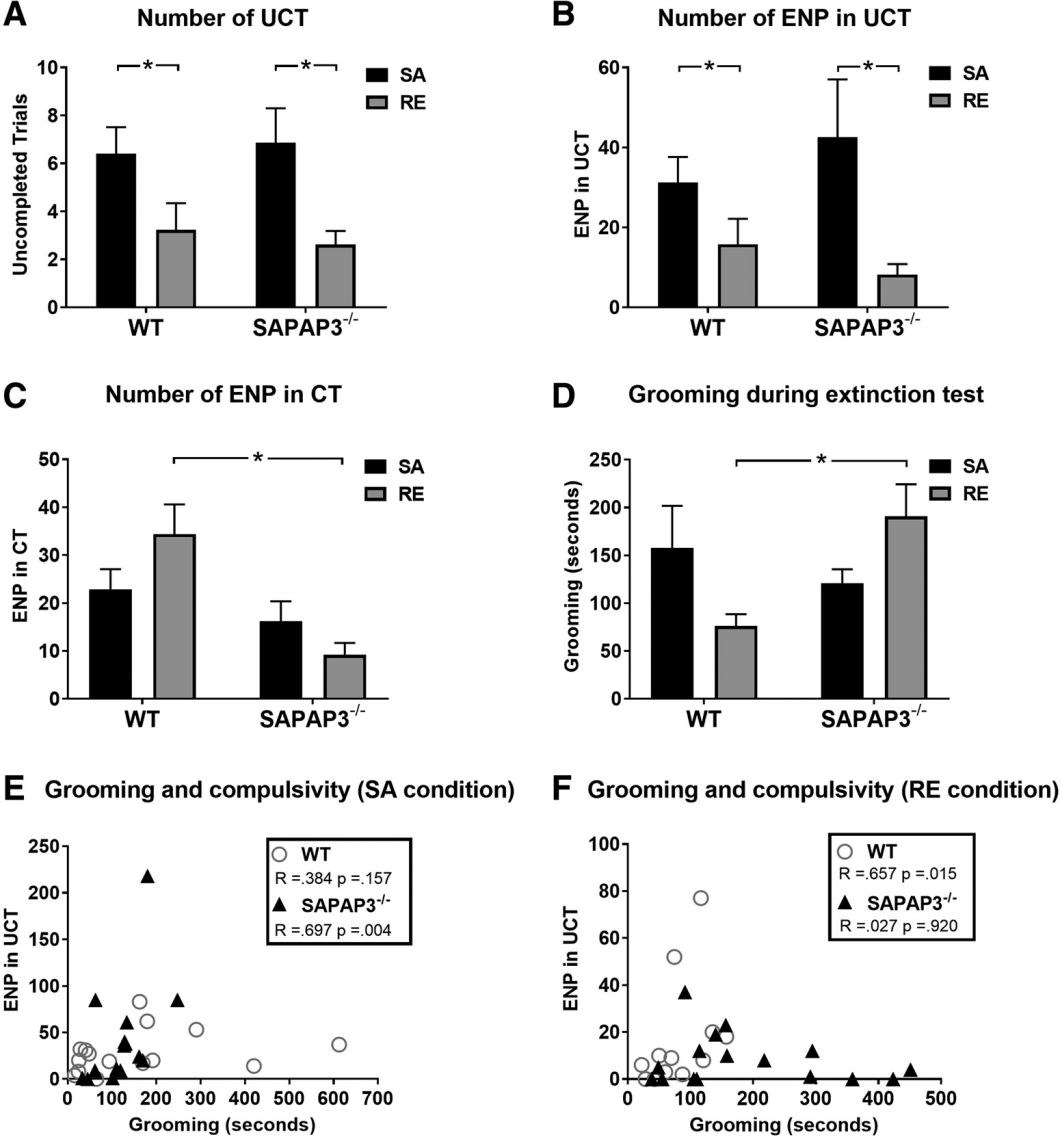
SAPAP3^-/-^ are not more compulsive than WT in the SA task. ***A***, SAPAP3^-/-^ do not show a general deficit in feedback processing, as compulsive responding during the extinction test (stage 4) of the SA paradigm was similar to that of normal WT controls. Both WT-SA and SAPAP3^-/-^-SA showed more UCTs than WT-RE and SAPAP3^-/-^-RE, respectively, confirming that the SA stage (stage 3) was effective in inducing compulsive-like responding. ***B***, ENPs in UCT, an important indicator of compulsivity, was similar between SAPAP3^-/-^ and WT mice that underwent SA, suggesting that genetic deletion of SAPAP3 does not potentiate SA-induced compulsivity. ENP in UCT were increased in WT-SA and SAPAP3^-/-^-SA compared with WT-RE and SAPAP3^-/-^-RE, respectively, confirming that the SA stage was effective. ***C***, A genotype difference was found in the number of ENP in CTs. During RE, SAPAP3^-/-^ showed reduced numbers of ENP in CT, indicative of rapid extinction learning or altered vigor for obtaining rewards. ***D***, During the extinction test (stage 4), SAPAP3^-/-^-RE groomed significantly more than WT-RE. In contrast, grooming was similar between genotypes after SA. ***E***, ***F***, During the extinction test (stage 4), a positive correlation was observed between grooming and ENP in UCT for SAPAP3^-/-^-SA but not for SAPAP3^-/-^-RE. WT mice showed a positive correlation between grooming and ENP in UCT in the regular-extinction condition but not following SA. The average duration of the extinction test varied between 39 and 41 min (SAPAP3^-/-^: SA =15, RE = 16; WT: SA = 17, RE = 13). Data are expressed as mean ± SEM. *p < 0.05.

Analysis of compulsive responses of WT-SA (*n* = 17) and WT-RE (*n* = 13) revealed that the SA procedure induced significantly more UCT compared with RE (*U* = 60.5, *p* = 0.035; WT-SA: 6.4 ± 1.1, WT-RE: 3.2 ± 1.1; Fig. 4*A*), most pronounced during the first two blocks of the test session [first block: χ^2^(1) = 12.109, *p* = 0.001; WT-SA: 1.6 ± 0.42, WT-RE: 0.0 ± 0.0 (no UCTs); second block: χ^2^(1) = 4.498, *p* = 0.034; WT-SA: 1.6 ± 0.37, WT-RE: 0.69 ± 0.36]. While there was no difference between WT-SA and WT-RE in the overall number of ENP in CT (Fig. 4*C*), analysis revealed more ENP-UCT of WT-SA compared with WT-RE (*U* = 56.0, *p* = 0.022; WT-SA: 31.2 ± 6.4, WT-RE: 15.8 ± 6.4; Fig. 4*B*). This suggests that both markers of compulsive-like behavior, UCT and ENP-UCT, are increased following the SA procedure.

### Compulsive- like responding in SAPAP3^-/-^-SA versus SAPAP3^-/-^-RE

Analysis of the response pattern of SAPAP3^-/-^ during the final extinction test revealed a significantly increased number of UCT in SAPAP3^-/-^-SA (*U* = 52.0, *p* = 0.006; SAPAP3^-/-^-SA: (*n* = 15) 6.9 ± 1.4, SAPAP3^-/-^-RE: (*n* = 16) 2.6 ± 0.56; Fig. 4*A*), specifically at the beginning of the session (first block of 10 trials: χ^2^(1) = 6.691, *p* = 0.010; SAPAP3^-/-^-SA: 1.9 ± 0.67, SAPAP3^-/-^-RE: 0.19 ± 0.10). Our results also show more ENP of SAPAP3^-/-^-SA compared with SAPAP3^-/-^-RE (*U* = 47.0, *p* = 0.004, SAPAP3^-/-^-SA: 58.8 ± 14.7, SAPAP3^-/-^-RE: 17.4 ± 4.8). Importantly, this difference was due to increased numbers of ENP in UCT in SAPAP3^-/-^-SA compared with SAPAP3^-/-^-RE (*U* = 53.0, *p* = 0.008; SAPAP3^-/-^-SA: 42.4 ± 14.5, SAPAP3^-/-^-RE: 8.2 ± 2.6; Fig. 4*B*), as no difference was detected in the performance of ENP in CT (Fig. 4*C*). These results confirm that decreased general feedback processing, induced by SA, reliably increases compulsive-like behaviors in SAPAP3^-/-^.

### Compulsive responding of SAPAP3^-/-^ versus WT

Comparison between SAPAP3^-/-^-SA and WT-SA as well as SAPAP3^-/-^-RE and WT-RE show similar numbers of UCT during the extinction test (χ^2^(1) = 0.111, *p* = 0.739; Fig. 4*A*). Further analysis revealed that ENP-UCT were not different between genotypes (χ^2^(1) = 0.872, *p* = 0.351; Fig. 4*B*). Taken together, these results suggest that SAPAP3^-/-^ in comparison to WT controls do not show excessive, compulsive-like behavior in the SA paradigm, neither under conditions of RE nor when general feedback processing is diminished by SA.

Interestingly, we found a difference in the number of ENP in CT between SAPAP3^-/-^ and WT (χ^2^(1) = 11.490, *p* < 0.001). Analysis showed more ENP in CT for WT than SAPAP3^-/-^ in the RE condition (*U* = 27.5, *p* < 0.001; WT-RE: 34.4 ± 6.2; SAPAP3^-/-^-RE: 9.2 ± 2.5) but not following SA (*U* = 95.5, *p* > 0.05). As numbers of CT and no-poke trials were similar between genotypes, this result may reflect a difference in extinction learning or reduced response vigor of SAPAP3^-/-^ (Fig. 4*C*).

### Grooming and anxiety in SAPAP3^-/-^ compared with WT

Grooming was scored during the extinction test. Results showed that SAPAP3^-/-^ generally groomed more than WT throughout the test [χ^2^(1) = 4.516, *p* = 0.034; SAPAP3^-/-^: 156.9 ± 19.4 s, WT: 119.9 ± 25.1 s]. Genotype differences in grooming were most apparent in the RE condition (*U* = 45.0, *p* = 0.01; SAPAP3^-/-^: 190.8 ± 33.4, WT: 76.2 ± 12.3 s), and were not present following SA (*U* = 107.0, *p* > 0.05; Fig. 4*D*).

Overall, a positive Spearman’s rank-order correlation was observed between grooming of all animals and ENP in UCT (*r*_(59)_ = 0.320, *p* = 0.014). Separated analysis for grooming of SAPAP3^-/-^ and WT in the RE and SA condition was performed. This analysis showed a positive Spearman’s rank-order correlation between the total duration of grooming and the number of UCT (*r_s_*_(13)_ = 0.779, *p* = 0.001) of SAPAP3^-/-^-SA, but not WT-SA. Furthermore, a positive correlation between duration of grooming and ENP in UCT (*r_s_*_(13)_ = 0.697, *p* = 0.004, Bonferroni corrected *p* = 0.0125) was observed in SAPAP3^-/-^-SA (Fig. 4*E*). In the RE condition, there was no significant correlation between duration of grooming and ENP in UCT following Bonferroni correction (*p* = 0.0125; Fig. 4*F*). Together, these results suggest a divergent grooming pattern between genotypes. In SAPAP3^-/-^, grooming was associated with compulsive-like behavior following SA, which was not observed in WT.

**Figure 5.**
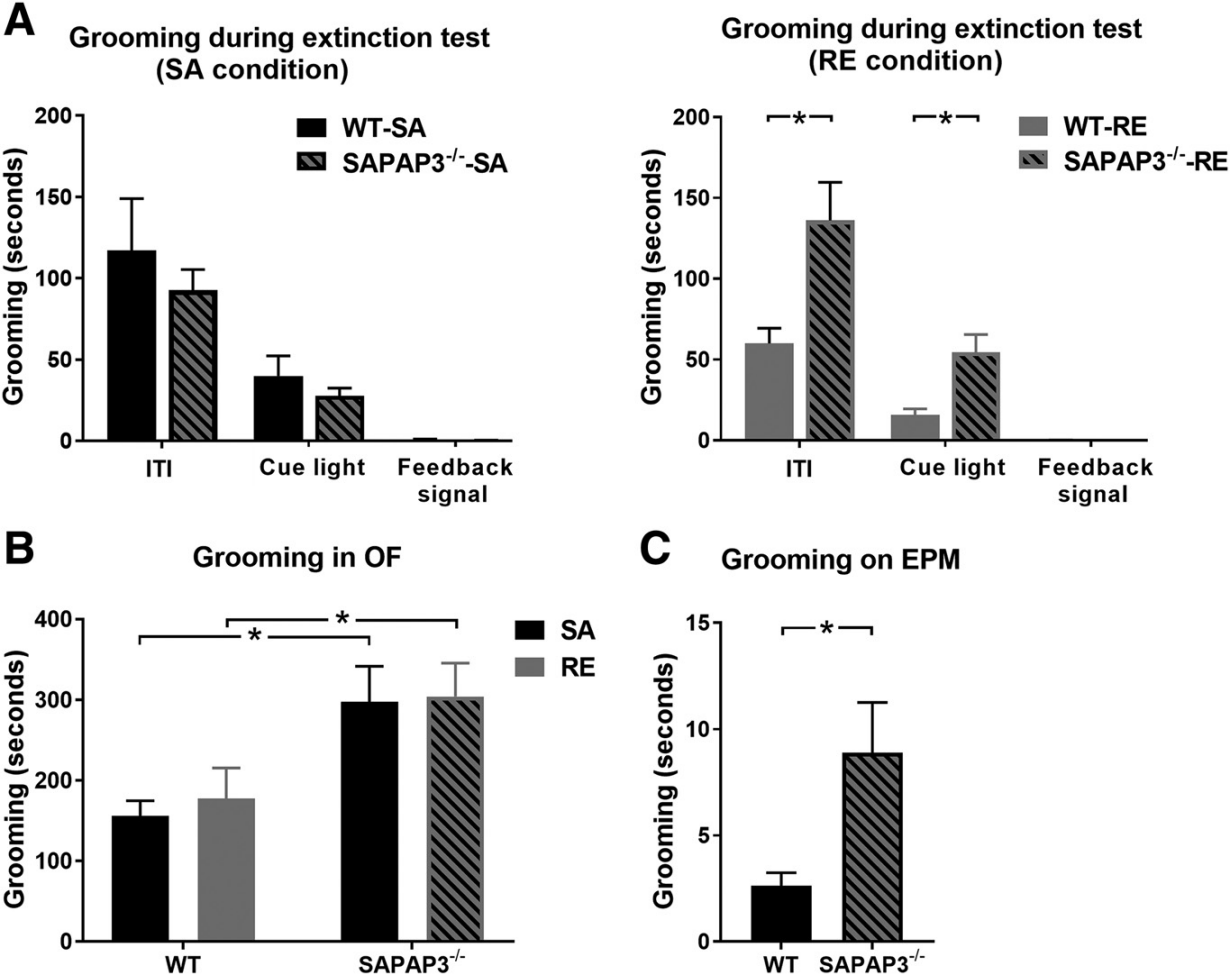
SAPAP3^-/-^ groom significantly more than WT in various contexts. ***A***, During the extinction test (stage 4), grooming is significantly higher in SAPAP3^-/-^ compared with WT in the RE condition, both during the ITI and during illumination of the cue light (indicator that an operant response is required). However, grooming during cue light presentation was not correlated with overall task performance (data not shown), suggesting that attention to behaviorally relevant cues was not diminished in SAPAP3^-/-^. ***B***, Additionally, grooming was scored in the open field (OF). Results show increased grooming behavior of SAPAP3^-/-^ compared with WT. ***C***, SAPAP3^-/-^ and WT were exposed to a 5-min test on the elevated plus maze (EPM). SAPAP3^-/-^ spent more time grooming than WT mice on the EPM; RE, average duration extinction test 39–41 min; OF, test duration 60 min; EPM, test duration 5 min (SAPAP3^-/-^: SA = 15, RE = 16; WT: SA = 17, RE = 13). Data are expressed as mean ± SEM. *p < 0.05.

Closer inspection of grooming behavior during task episodes of ITI, nose-poke light and feedback signal presentation revealed that grooming of SAPAP3^-/-^ was specifically increased in the RE condition during cue light presentation (χ^2^(1) = 6.694, *p* = 0.01; SAPAP3^-/-^: 54.7 ± 10.8 s, WT: 15.9 ± 3.8 s) and ITI (χ^2^(1) = 6.923, *p* = 0.009; SAPAP3^-/-^: 136.1 ± 23.6 s, WT: 60.1 ± 9.2 s: Fig. 5*A*). Grooming during feedback signal presentation occurred only incidentally and was not different between genotypes. Grooming during nose-poke light presentation might limit attentional resources for task performance, but could also reflect decreased commitment to the task.

In addition to task-related grooming, general grooming behavior of SAPAP3^-/-^ and WT was scored in an open-field session that lasted 1 h. A Kruskal–Wallis test showed significantly increased grooming of SAPAP3^-/-^ compared with WT in SA (χ^2^(1) = 5.570, *p* = 0.018; SAPAP3^-/-^-SA: 297.3 ± 44.5 s, WT-SA: 156.0 ± 78.1 s) and RE (χ^2^(1) = 5.402, *p* = 0.020; SAPAP3^-/-^-RE: 304.1 ± 41.6 s, WT-RE: 177.5 ± 37.9 s), confirming the compulsive-like phenotype of SAPAP3^-/-^ (Fig. 5*B*).

Additionally, 18 SAPAP3^-/-^ and 18 WT were subjected to a 5-min test on the EPM. SAPAP3^-/-^ and WT spent similar amounts of time exploring the open (*U* = 116.0, *p* = 0.146) and closed arms of the maze (*U* = 103.0, *p* = 0.062), suggesting that this cohort of young adult SAPAP3^-/-^ did not yet develop increased anxiety symptoms. During this test, SAPAP3^-/-^ were observed with exaggerated grooming compared with WT (*U* = 96.0, *p* = 0.036; SAPAP3^-/-^: 8.9 ± 2.4 s; WT: 2.6 ± .62 s; Fig. 5*C*). Overall, grooming behavior of SAPAP3^-/-^ and WT was likely influenced by genotype, task conditions and environmental requirements, as SAPAP3^-/-^ groomed consistently more than WT, but grooming measures in various contexts were not correlated.

## Discussion

Here, we investigated whether the compulsive phenotype observed in a mouse model for OCD, the compulsively grooming SAPAP3^-/-^ (Welch et al., 2007), is associated with a deficit in feedback processing. First, we implemented the SA task in mice, previously established for rats only. Our results show that mice behaved similarly to rats after being subjected to simulation of feedback deficiency (SA): compared with mice that experienced a “regular” extinction, they displayed more UCTs and, importantly, more ENPs, specifically during UCTs (ENP in UCT). The latter is used as a direct measure of compulsive responding in the SA task (Joel, 2006). Herewith, we validate the SA task to study deficient feedback processing in mice.

Next, we compared the behavior of SAPAP3^-/-^ and their WT littermates in the SA task. SAPAP3^-/-^ did not display more UCT or ENP-UCT after SA-induced feedback deficiency than WT. Thus, contrary to our initial hypothesis, SAPAP3^-/-^ compulsive responding was not enhanced compared with WT, suggesting that different mechanisms underlie compulsive grooming and compulsive responding in the SA task.

### SA in mice and rats

To understand compulsivity in the SA task, it is important to consider how different measures of compulsivity are related. In the SA stage (stage 3), the signal is presented without reward and nose-poke holes are inaccessible, thereby reducing the association between signal and reward, presumably without affecting response-outcome associations. By keeping response-outcome associations intact, it is possible to investigate the effect of ‘distorted’ feedback-processing on behavior. We focused our analysis on the most prominent indicators of compulsive behavior, as described by previous SA studies: UCT (correct responses not followed by attempts to collect reward) and ENP in UCT (perseveration of nose pokes in trials with correct responses not followed by attempts to collect reward). Response perseveration (ENP) is independent of trial structure because ENP are performed before attempting (or not attempting) reward collection. Thus, with more UCT there are not necessarily more ENP. We focused on ENP in UCT, instead of reporting proportions of ENP per UCT, because the relation of these essentially independent outcome measures does not necessarily inform about an animal’s degree of compulsivity. For example, when comparing an animal with one ENP in one UCT and an animal performing ten ENP in ten UCT, the latter one clearly displays more compulsive behavior revealed by the absolute numbers.

Generally, the measures for compulsivity in the SA task (UCT and ENP in UCT) likely represent behavior that is driven by behavioral uncertainty about current signal-outcome associations. In the SA condition, subjects learn that response feedback no longer signals reward availability (induction of feedback deficiency). When they subsequently experience the absence of reward after a CT in the final extinction test, they may experience the impulse to respond repeatedly when given the opportunity to perform this previously reinforced operant response.

We can conclude from experiment 1 that the SA condition induces compulsive responding in “normal” mice, marked by a significant increase of UCT and ENP-UCT, which is similar to effects generally observed in the rat model of SA (Joel, 2006; Albelda and Joel, 2012). We noticed that mice required more training sessions than rats to acquire responding for food, possibly reflecting differences in the species’ learning abilities or effects of different response manipulanda (i.e., we used nose-poke holes whereas rats are usually trained with levers). However, performance of rats and mice in the final extinction test are comparable regarding the numbers of CT, no-poke trials, UCT, and ENP in UCT. Together, these findings across species provide further support for the hypothesis that relevant feedback cues are regulators of behavior and that attenuation of the incentive salience of these signals may cause difficulty in preventing behavior from becoming compulsive.

### Comparison of SAPAP3^-/-^ and WT in the SA task

In experiment 2, we trained SAPAP3^-/-^ and WT in the SA task to determine whether SAPAP3^-/-^ show enhanced compulsivity compared with WT in the final extinction test. Importantly, our results demonstrate that the number of UCT and ENP-UCT were similarly increased in SAPAP3^-/-^ and WT that underwent the SA procedure compared with SAPAP3^-/-^ and WT that experienced RE. The absence of genotype differences in these compulsivity measures does not fulfill our prediction of potentiated compulsive responding of SAPAP3^-/-^ under conditions of decreased feedback processing or during RE. Nonetheless, this finding provides important insight into the nature of compulsivity as it implies that different neurobiological mechanisms might independently lead to different aspects of compulsivity.

### Comparison of grooming and compulsive responding in the SA task

SAPAP3^-/-^ exhibited enhanced grooming throughout the final extinction test compared with WT, confirming the exaggerated grooming phenotype of the SAPAP3^-/-^ model (Welch et al., 2007). However, this was due to the large differences in grooming in the RE condition, which suggests an interaction between grooming and task-related behavior in both genotypes. Grooming in SAPAP3^-/-^ was consistently increased over WT values in all provided experimental environments (operant box during SA paradigm, OF, EPM). Similar to previous studies, grooming scores in different task conditions, such as the SA task, OF, and EPM, were uncorrelated, suggesting that the degree to which individual SAPAP3^-/-^ display this compulsive-like behavior is highly variable (Pinhal et al., 2018; Manning et al., 2019; van den Boom et al., 2019). Consistently, grooming is indeed known to be influenced strongly by emotional factors and environmental conditions (Kalueff et al., 2016).

A remarkable finding in this respect was that the extinction test (in SA-exposed mice) was the only condition in which grooming did not differ between SAPAP3^-/-^ and WT. This was accompanied by a positive correlation between grooming and task-induced compulsivity measures for SAPAP3^-/-^ in the SA condition. This indicates that the impact of this specific task phase on spontaneous (grooming) behavior is dependent on both task condition (SA or RE) and genotype. This is in contrast to the task-induced compulsive responding which exclusively depends on the task condition and is in conflict with the strong genotype dependence of spontaneous grooming in all other test conditions. Therefore, we do not take this as evidence for the existence of a relation between both forms of compulsivity and conclude that the SAPAP3^-/-^ genotype resulting in compulsive grooming does not result in an increased susceptibility for compulsivity induced by feedback uncertainty.

### Comparison with tests using feedback signals in OCD

The results of our study are important in light of the findings of a previous study examining the course of feedback-dependent learning in human OCD patients. During initial trial-and-error learning, in which behavioral responses needed to be updated by external feedback, OCD patients were observed to exhibit response deficits (Nielen et al., 2009). At a later point in training, however, OCD patients showed similar performance to controls. Thus, potentially decreased employment of external feedback signals might cause only transient learning deficits, that are less important for behavioral outcomes than OCD-related compulsive symptoms and could reflect decreased task engagement caused by altered processing of appetitive rewards, a condition that was also reported for human OCD patients (Figee et al., 2011; Marsh et al., 2015). Although the study by Nielen et al. (2009) provides valuable insight into feedback processing in patients, the exact point at which external feedback becomes less important for behavioral choices cannot be determined with this paradigm, nor is external feedback directly modulated. Future studies may employ the methodology of the SA task to further investigate processing of feedback signals related to obsessive-compulsive behaviors in OCD patients.

### Cognitive dysfunction and compulsivity in SAPAP3^-/-^

The question that follows from our findings is whether our conclusion that different neurobiological mechanisms may independently lead to different aspects of compulsivity (and ultimately to a compulsive-like phenotype; Figee et al., 2013), also applies to other cognitive dysfunctions that have been proposed to be associated with compulsive behaviors. Evidence is accumulating that cognitive inflexibility and an imbalance between goal-directed and habitual behavior may be present in the SAPAP3^-/-^ model for OCD (Manning et al., 2019; van den Boom et al., 2019; Ehmer et al., 2020). The relation between the dysfunction, compulsive grooming, and the genetic deletion, however, seems to be complex: neither cognitive inflexibility nor deficient habit formation were correlated to compulsive grooming. Taken together, this implies that the genetic defect may result in excessive grooming, impaired reversal learning, and deficient habit formation (in appetitive learning), but that these effects are not obviously linked to one another and possibly involve more complex or different neurobiological mechanisms.

### Limitations

While the SA paradigm provides information about mechanisms of general feedback processing, internal feedback specifically related to compulsive behavior has not yet been investigated. It can thus not be excluded that different task conditions have an effect on a potential deficiency in processing of internal feedback signals related to compulsive grooming in SAPAP3^-/-^. Finally, our study comprised only positive response feedback for an appetitive learning condition. Future investigation on processing of aversive feedback signals, or processing of feedback signals with positive or negative valence on avoidance behaviors is indicated.

In summary, the SA task simulates feedback deficiency, which is hypothesized to contribute to compulsive symptoms in psychiatric disorders such as OCD. The purpose of the current study was to implement the SA task in mice and to evaluate the SA-induced behavior of SAPAP3^-/-^ compared with their normal littermates. The performance of SAPAP3^-/-^ mutants in the SA task did not show a potentiation of compulsivity, suggesting that these two models of compulsivity do not share a common neuronal pathology and that different types of compulsivity exist in parallel.

## Synthesis

Reviewing Editor: Jorge J. Palop, Gladstone Institutes and UCSF Decisions are customarily a result of the Reviewing Editor and the peer reviewers coming together and discussing their recommendations until a consensus is reached. When revisions are invited, a fact-based synthesis statement explaining their decision and outlining what is needed to prepare a revision will be listed below. The following reviewer(s) agreed to reveal their identity: Eric Burguiere.

Thank you so much for your great comments and reviewing this manuscript. We all agree with the decision “Revise and Re-Review”. Please find below the synthesis, which includes your comments and a few extra clarifications. Please let me know within 24 hours if you agree or want to modify the synthesis statement.

SYNTHESIS.

This article aims at investigating one of the cognitive function that may lead to compulsivity, namely the processing of feedback information. For this purpose, the authors proposed to adapt the signal attenuation (SA) developed originally in rats to mice. This task consists of mimicking the degradation of feedback processing signal presented after an operant response. As a result, they first adapted and demonstrated that the SA task can be implemented in mice. Second, they tested the hypothesis that the Sapap3 knockout mouse model of obsessive-compulsive disorder (OCD), which expresses compulsive and excessive grooming, may show enhanced compulsive-like behavior in the SA task. In this second part, the authors could not find differences in compulsive-like instrumental behavior in Sapap3-KO mice compared to WT mice and conclude that this type of compulsive behavior may have different mechanistic origin than compulsive grooming.

The current manuscript advances a better understanding of the different and non-overlapping types compulsive responding with their genetic underpinnings, including the SAPAP3 KO mouse model. This is an interesting conceptual paper that has the originality to challenge a hypothesis that has been proposed for understanding the functional deficit observed in OCD patients. However, I have concerns on different parts of the manuscript concerning methodology, conceptual framework, and interpretation of some results that should be addressed:

1. In their introduction, the authors mentioned (l.89) “then compulsivity in the SA task should be exacerbated in animal models for OCD (which already display compulsivity prior to SA induction of compulsivity)”. I think that the authors should already distinguish the possible different types of compulsivity which are engaged either in grooming or during the SA-task. Indeed, the conceptual link between compulsive grooming and a higher susceptibility to the SA-task is not straightforward, so their hypothesis could be more didactically formulated.

2. On the methods section, the authors mentioned that “a speaker was attached to each chamber that produced tones with 80 dB and 2.8 kHz.” These values are a bit surprising, knowing that mice have a very degraded hearing at these “low” frequencies. This has been documented in several studies (e.g. Ouagazzal AM, Reiss D, Romand R. Effects of age-related hearing loss on startle reflex and prepulse inhibition in mice on pure and mixed C57BL and 129 genetic background. Behav Brain Res. 2006 Sep 25;172(2):307-15). Can the authors explain why they used such low values? Did they try to replicate experiment with higher frequencies? Can they guarantee that Sapap3 KO are perceiving as well as WT these signal, especially since we are at extreme range for mice hearing?

3. At l.138: “in two successive sessions to proceed to training stage 2”. Can the authors specify how long a session is? If there are significant difference between session duration across animal, did they look at the rate frequency to characterize compulsive poking rather than absolute number?

4. At l. 196, the authors described: “Further, we calculated the percentage of ENP-UCT from the total of ENPs and the number of ENP per UCT as outcome measures, because animals differed in the display of UCTs”. This is not the case in the results showed in the figures; we do not see proportion but absolute amount. I think it’s indeed rather important to report proportion to disambiguate if it is perseveration on the response per se rather than higher number of UCT leading to more ENP.

5. At l.230, the authors said that “C57Bl6-SA mice had more no-poke trials” during the final extinction protocol. This statement emphasizes a conceptual question I have about the SA task. Since the mice are exposed, during the SA stage, to the same environment but with no access to the reward despite magazine entries, why can’t we assume that there is some extinction happening already during this SA stage? This seems to be proven by the no-poke trials observed in the extinction stage, and thus the higher number of UCT could also be a consequence of this partial extinction rather than feedback signal degradation?

6. The results obtained in the SA task could be interpreted as the authors suggest with Sapap3 KO mice grooming compulsivity of different nature than compulsive-like ENP but the previous comments should be clarified and addressed. Indeed, as an alternative explanation, the protocol could provoke a longer extinction due to the SA stage, then this will lead to higher number of UCT which in turn would lead to higher number of ENP but, if not reported as a higher rate of ENP per UCT, is not related to compulsive-like behavior.

7. It is hard to tell, but it appears that the authors used a repeated measures analysis with a Friedman test for the non-parametric analysis but this is learning data across sessions/days and as such the data pts. are not independent from one another violating the repeated measures design assumption. Data distribution seems pretty normal, two factor ANOVA (session and genotype) might be the best way to assess the session and genotype effects in Fig. 3C and 3D.

8. Chi-square analysis (x2) appears multiple times in the results section without any description in the methods. For example, line 278, 282, 289, etc.

9. It is unclear if the SA data is averaged over all 3 sessions of 30 trials each or not. The direct comparison of this performance to only 1 session of 50 trials in the Extinction responding seems quite unequal (Figure 4). Please explain the data source more clearly and how direct comparisons of uncompleted trials, ENP, and grooming time are valid given the discreet differences in the numbers of trials in the SA and Extinction sessions.

10. Line 247, genotype matching on task performance. “Genotype comparison of training performance was not different between SAPAP3-/- and WT, confirming that both genotypes acquired the task equally.” Since animals were matched to task performance in order to compare compulsivity between the genotypes, it makes no sense to assess the genotype effect since animal were removed. Please remove the statistical analysis comparing the acquisition of the different training steps of the task given that the groups were purposely matched a priori to the analysis. Please indicate in the result section that matching was performed to clarify that the goal was not to assess a genotype difference.

11. Grooming assessments during competing behaviors. Distinct measurements of grooming time during the performance of other tasks reflects more about behavioral competition than simple compulsive grooming behavior, and when you consider the very small amounts of grooming time exhibited in the SA or RE tests as well as the EPM, the only true grooming difference seems to be in the Open Field with a longer testing time that provides the assessment of grooming periods without behavioral competition. It would be better to present Figure 5 data as % time grooming to highlight this caveat and include the total time of the test in the figure legend.

12. Compulsive responding as defined by ENP in UCT. Compulsive responding as defined only by the ENP in UCT seems a bit limiting in scope given that the data in Fig. 4 shows only 13% of trials (50 trials) as uncompleted, thus limiting the interpretation given that Saoap3 KO mice do not show more compulsive responding except in the completed trials for the RE groups.

13. Correlations with self-grooming. The restricted range of self-grooming times during the final extinction test limit the strength of the correlational analyses such that they are drastically affected by measures at the extremes. Consider removing as they do not add significant information to the data.

14. Line 209, the result subheadings “Training C57Bl6 mice” is not informative. Please use subheadings that are specific and informative and that capture the main conclusion of the result section.

14. Line 210, change “Fig. 2” to “Fig. 2A”.

15. Line 243, change “Training WT and SAPAP3-/-” to an informative subheading. Please consider to add “mice” to this title and through the manuscript.

16. Line 208 and 242, please remove experiment 1 and 2.

17. Figure 2, you may want to use WT-RE and WT-SA labels for Fig. 2. It is a bit confusing that wildtype mice are named “C57Bl6-SA” in Figure 2 and “WT-SA” in other figures.

18. Figure 3, consider to add the genotype to the key of the figure panels by changeing “SA” to “WT-WT” or “SAPAP3-/-SA”.

19. Line 262, change “Fig 3c” to “Fig.3C”.

20. Figure legends, indicate the n number of all genotypes in the figure legends.

21. The title seems too long and technical for the general audience. General audience does not know that the Sapap3 ko is a genetic model of obsessive-compulsive disorder. For example, “Evidence of distinct forms of compulsivity in the Sapap3 knockout mouse model of obsessive-compulsive disorder” would be more appealing and easier to understand.

## Author Response

We are grateful that the reviewers prompted us to improve and explain our work and hope to address all questions and concerns sufficiently:

1. In their introduction, the authors mentioned (l.89) “then compulsivity in the SA task should be exacerbated in animal models for OCD (which already display compulsivity prior to SA induction of compulsivity)”. I think that the authors should already distinguish the possible different types of compulsivity which are engaged either in grooming or during the SA-task. Indeed, the conceptual link between compulsive grooming and a higher susceptibility to the SA-task is not straightforward, so their hypothesis could be more didactically formulated.

Our ingoing hypothesis for this study was that compulsivity is a unitary and uniform phenomenon with an underlying pathology that may give rise to different expressions of compulsive behavior. Thus, we expected to find enhanced compulsivity in SAPAP3 knockout mice (genetic OCD model) in the SA paradigm, comparable to the finding of Sesia et al (2013) showing that compulsivity in the SA procedure is enhanced after repeated quinpirole administration (pharmacological OCD model). Nevertheless, we agree with reviewer that it is useful to mention the alternative hypothesis explicitly. Therefore, we added the following to the introduction text: “Alternatively, a variety of neural mechanisms might independently cause qualitatively different forms of compulsivity that do not potentiate each other. In this case, SA-induced compulsivity would not differ between SAPAP3-/- and WT.”. [Line 94-96]

2. On the methods section, the authors mentioned that “a speaker was attached to each chamber that produced tones with 80 dB and 2.8 kHz.” These values are a bit surprising, knowing that mice have a very degraded hearing at these “low” frequencies. This has been documented in several studies (e.g. Ouagazzal AM, Reiss D, Romand R. Effects of age-related hearing loss on startle reflex and prepulse inhibition in mice on pure and mixed C57BL and 129 genetic background. Behav Brain Res. 2006 Sep 25;172(2):307-15). Can the authors explain why they used such low values? Did they try to replicate experiment with higher frequencies? Can they guarantee that Sapap3 KO are perceiving as well as WT these signal, especially since we are at extreme range for mice hearing?

The reviewer is concerned about whether mice are able to detect the auditory component of the feedback signal and whether there may be differences in perception of it between genotypes.

1) First, in the present study, no difference in SA task performance was observed between SAPAP3-/- and WT, indicating that perception between genotypes did not differ.

2) Our behavioral setups are equipped with standard devices from Med Associates (St. Albans, VT, USA), and our lab has not experienced any difficulties with mice learning associations that are based on auditory stimuli produced by these devices at 2.8 kHz. Neither in SAPAP3-/- nor in WT this was described (van den Boom et al., 2019).

3) Another manufacturer of operant boxes for mice (other than Med Associates), routinely applies 3 kHz tones as auditory cues in their standardized behavioral tests (Campden Instruments, Cambridge, UK). Authors associated with this company report successful application of this tone frequency to signal trial start or as conditioned reinforcer (1-second presentation) (Horner et al., 2013).

4) In the present study, the auditory stimulus was always just one of the two components of a compound audio-visual stimulus that signaled reward availability. Therefore, behavioral responses were not exclusively reliant on auditory perception. Consequently, even if mice were to experience auditory perception difficulties, they could still rely on the visual component of the compound stimulus.

Horner AE, Heath CJ, Hvoslef-Eide M, Kent BA, Kim CH, Nilsson SR, Alsiö J, Oomen CA, Holmes A, Saksida LM, Bussey TJ (2013) The touchscreen operant platform for testing learning and memory in rats and mice. Nat Protoc. 8(10):1961-84. doi: 10.1038/nprot.2013.122.

van den Boom BJG, Mooij AH, Misevičiūtė I, Denys D, Willuhn I (2019) Behavioral flexibility in a mouse model for obsessive-compulsive disorder: Impaired Pavlovian reversal learning in SAPAP3 mutants. Genes Brain Behav. 18(4): e12557. doi: 10.1111/gbb.12557.

3. At l.138: “in two successive sessions to proceed to training stage 2”. Can the authors specify how long a session is? If there are significant difference between session duration across animal, did they look at the rate frequency to characterize compulsive poking rather than absolute number?

1) The reviewer requests information about session duration. The different stages of the SA task were programmed with a trial structure, wherein time windows existed during which the animals were required to respond.

In the magazine training (stage 1), an animal had a max of 15 seconds to obtain a reward. As soon as the reward was retrieved, the trial ends and a new trial is started after a variable inter-trial interval (ITI). Thus, trial length depended on response latency. Consequently, a magazine-training session (stage 1) could theoretically last between 15.5 to 27 minutes. In the following nose-poke training (stage 2), animals needed to make an response within 15 seconds of cue-light presentation and collect the reward within 10 seconds to complete a trial. Together with the variable ITI, this could lead to a session length between 16 to 31.5 minutes for NP30 training. NP50 training and the final extinction test could last between 26.7 to 52.5 minutes each. Session duration of SA sessions (stage 3) could theoretically vary between 15.5 to 24.5 minutes.

2) The reviewer asks whether there is a difference in session length between animals. To address this question, we ran an additional analysis on the duration of the final extinction test (stage 4). A two-way ANOVA of session duration was performed with genotype (SAPAP3-/-, WT) and condition (SA, RE) as main factors. This analysis revealed a main effect of condition (F(1,57)= 39.39, p < .001, η2= .40), but no effect of genotype (F(1,57)= .487, p=.488) or genotype-condition interaction (F(1,57)= .012, p=.912). Post-hoc student’s t-tests showed that SA animals required more time to finish the extinction test in both genotypes (SAPAP3-/--SA: 40.9{plus minus}.21min, SAPAP3-/--RE: 39.1{plus minus}.44min, p=.001; WT-SA: 40.7{plus minus}.21min, WT-RE: 38.8{plus minus}.21min, p<.001). Thus, SAPAP3-/- and WT did not differ in extinction test duration, rendering additional analysis using response rate unnecessary (see figure below).

4. At l. 196, the authors described: “Further, we calculated the percentage of ENP-UCT from the total of ENPs and the number of ENP per UCT as outcome measures, because animals differed in the display of UCTs”. This is not the case in the results showed in the figures; we do not see proportion but absolute amount. I think it’s indeed rather important to report proportion to disambiguate if it is perseveration on the response per se rather than higher number of UCT leading to more ENP.

We thank the reviewer for identifying an error in referencing between statistical reporting in the manuscript and its representation in the figure. We now corrected this error [Line 239]. Importantly, we thought more about the best representation of our results and we decided to remove the “ENP per UCT” measure from the manuscript [previous Line 196-197; 236-239], and instead focus only on “ENP in UCT”. The reason for this is that we believe this makes the complex outcome measures of this task more comprehensible. Previously, we calculated “ENP per UCT” to reveal how many ENP animals displayed relative to the specific number of UCT. However, this results in a mixed variable in which both independent measures of compulsivity are expressed as relative to one other. In other words, this does not necessarily provide information about an animal’s level of compulsivity. For example, an animal with 1 ENP in 1 UCT receives the same value as an animal showing 10 ENP in 10 UCT, while the latter one clearly displays more compulsivity revealed by the absolute numbers. Furthermore, response perseveration, reflected in the number of ENP, is independent of trial structure because they are performed prior to attempting- or not attempting reward collection. Thus, with more UCT there are not necessarily more ENP (and ENP are also observed in CT, see Fig. 4C). We hope the reviewer follows our line of argumentation and agrees ENP in UCT provide sufficient information about compulsivity. We believe that this marginal simplification will make it easier for readers to understand our results.

5. At l.230, the authors said that “C57Bl6-SA mice had more no-poke trials” during the final extinction protocol. This statement emphasizes a conceptual question I have about the SA task. Since the mice are exposed, during the SA stage, to the same environment but with no access to the reward despite magazine entries, why can’t we assume that there is some extinction happening already during this SA stage? This seems to be proven by the no-poke trials observed in the extinction stage, and thus the higher number of UCT could also be a consequence of this partial extinction rather than feedback signal degradation?

We appreciate that the reviewer is conceptually probing the SA task and thinks along. The point raised is a good one. The reviewer is correct in that the idea underlying the application of the SA stage indeed produces extinction (or weakening) of the signal-reward association (stage 3; termed feedback signal degradation), by placing the animal in the context in which a specific signal was paired with reward delivery previously and presenting the signal without reward. Our approach was identical to the one performed in many previous studies in which “rats underwent an extinction of the classical contingency between the stimulus and the food” (established in stage 1) (e.g., Joel & Avisar, 2001). Notably, during this process, there is no extinction of the operant response, because nose-poke holes are covered (made inaccessible) in order to prevent this form of extinction. Only in stage 4, the final extinction test, extinction of operant responses is possible, because the animals have access to the poke holes (experienced in absence of reward delivery following a correct response). In theory of the SA paradigm this causes increased compulsivity in SA-exposed animals combined with a progressive decrease of completed trials and progressive increase of no-poke trials in all animals.

But the reviewer is correct that, in practice, we cannot know with absolute certainty if the mechanism is due to a pure “extinction of the feedback signal” or a “generally longer extinction” in the SA animals. However, we would like to argue that 1) neither our work nor the results of Joel & Avisar (2001; see figure below) show a significant increase of UCTs across time (blocks) in the RE groups in the test session (our Fig 2D and figure presented below). 2) In both, SA and RE condition, occurrence of UCTs is relatively even distributed throughout the session (presented in blocks), making it more likely that animals generally refrain from responding rather than replacing CTs with UCTs during extinction (indicating that there is no a sequential switch from CTs to UCTs). 3) We know that the SA procedure produces increased compulsive-like behavior in form of ENP in UCT (which is a unique result of the SA procedure and is not achieved in the RE group, not even as a trend) and UCTs.

Joel D, Avisar A (2001) Excessive lever pressing following post-training signal attenuation in rats: A possible animal model of obsessive compulsive disorder? Behavioural Brain Research 123: 77-87.

6. The results obtained in the SA task could be interpreted as the authors suggest with Sapap3 KO mice grooming compulsivity of different nature than compulsive-like ENP but the previous comments should be clarified and addressed. Indeed, as an alternative explanation, the protocol could provoke a longer extinction due to the SA stage, then this will lead to higher number of UCT which in turn would lead to higher number of ENP but, if not reported as a higher rate of ENP per UCT, is not related to compulsive-like behavior.

We agree that in order to best interpret SAPAP3-/- compulsivity in the SA task, it is important to understand how different compulsivity measures are related. It is correct that in the SA stage (stage 3) only the signal, but neither rewards nor operant manipulanda are accessible to the animal. This way, the association between signal and reward is reduced/extinguished, presumably without affecting the animal’s response-outcome association. Thus, by keeping the response-outcome association intact, it is possible to investigate the effect of ‘distorted’ feedback processing on behavior. Similar to previous studies, we focused our analysis on two indicators of compulsivity that were described as effects of the feedback manipulation: UCT (correct response not followed by an attempt to collect the reward) and ENP (perseveration of responses). These indicators are not necessarily dependent on one another (animals may or may not display ENP in UCT). The points made in our answer to question 5 apply here as well (see above).

As our goal was to compare different expressions of compulsivity, we examined the most prominent compulsive-like behavior in this task: the amount of ENP in UCT (perseveration of operant responses not followed by an attempt to collect reward). Our results showed no difference between SAPAP3-/- and WT in neither ENP in UCT (Fig. 4B), nor the total number of UCT (Fig. 4A). Comparing the distribution of ENP in the total number of UCT (ENP per UCT) would not change the interpretation of our results significantly.

7. It is hard to tell, but it appears that the authors used a repeated measures analysis with a Friedman test for the non-parametric analysis but this is learning data across sessions/days and as such the data pts. are not independent from one another violating the repeated measures design assumption. Data distribution seems pretty normal, two factor ANOVA (session and genotype) might be the best way to assess the session and genotype effects in Fig. 3C and 3D.

The reviewer suggests the use of ANOVAs instead of the non-parametric Friedman tests for the analysis of magazine entries of SAPAP3-/- and WT during SA (stage3, Fig. 3C and 3D). Normality testing for this data did not identify a significant deviation from normal distribution (Shapiro-Wilk Test >.05) and, thus, allowed for the requested parametric tests.

We now provide the following analysis in the text:

“The following signal attenuation procedure (stage 3, simulation of feedback deficiency) was analyzed with a two-factor ANOVA (session and genotype). Results show a significant effect of session (F(2,60) = 32.267, p < .001), but no session-genotype interaction or genotype effect. ANOVAs per genotype confirmed that WT-SA magazine entries declined across three successive SA sessions (F(2, 32) = 38.6, p < .001; 1st session SA 25.8{plus minus}1.3; 2nd session 20.5{plus minus}1.1; 3rd session 17.0{plus minus}0.9), suggesting effective attenuation of the feedback signal (Fig.3C). In SAPAP3-/--SA, effective attenuation of the feedback signal was also demonstrated by decreasing magazine entries across SA training sessions (F(2, 28) = 7.09, p =.003; 1st session SA 21.8{plus minus}1.4; 2nd session 18.5{plus minus}1.4; 3rd session 16.1{plus minus}1.4)(Fig.3D).” [Line 250-257]

As for the other part of this comment, we are not sure whether we understand completely: Repeated-measures analysis is by definition based on data points that are not independent from one another. Thus, dependent data points do not violate a repeated-measures design assumption.

8. Chi-square analysis (x2) appears multiple times in the results section without any description in the methods. For example, line 278, 282, 289, etc.

Indeed, several comparisons were performed with Kruskal-Wallis H test, employing the chi-square statistic. In response to the reviewer’s comment, we now improved our description of the test in the Methods section:

“Kruskal-Wallis H tests provided the chi-squared statistic for analyzing non-parametric data with more than two independent samples, while Mann-Whitney U tests were employed for comparison of two independent samples. Repeated-measures ANOVA was employed when data was presumably normally distributed.” [Line 202-205]

9. It is unclear if the SA data is averaged over all 3 sessions of 30 trials each or not. The direct comparison of this performance to only 1 session of 50 trials in the Extinction responding seems quite unequal (Figure 4). Please explain the data source more clearly and how direct comparisons of uncompleted trials, ENP, and grooming time are valid given the discreet differences in the numbers of trials in the SA and Extinction sessions.

At seems as if there was confusion between different stages of the SA paradigm: During the SA stage of the experiment (stage 3), half of the animals underwent three successive SA sessions. Animals that were subjected to stage 3 are labeled with their respective genotyped followed by “-SA”. Results of the three SA sessions are shown in Fig. 3C for “WT-SA” and Fig.3D for “SAPAP3-/--SA”.

In the following extinction test (stage 4), all animals underwent 50 trials. For the analysis of the extinction test, we then compare behavior of animals that were previously subjected to SA (WT-SA, SAPAP3-/--SA) and those that experience “regular” extinction (RE) without prior feedback manipulation (WT-RE, SAPAP3-/--RE).

10. Line 247, genotype matching on task performance. “Genotype comparison of training performance was not different between SAPAP3-/- and WT, confirming that both genotypes acquired the task equally.” Since animals were matched to task performance in order to compare compulsivity between the genotypes, it makes no sense to assess the genotype effect since animal were removed. Please remove the statistical analysis comparing the acquisition of the different training steps of the task given that the groups were purposely matched a priori to the analysis. Please indicate in the result section that matching was performed to clarify that the goal was not to assess a genotype difference.

The reviewer points out correctly that genotype matching was performed based on training performance during nose-poke acquisition (stage 2), as we aimed for assessing genotype differences during the extinction test only. However, after matching, animals were randomly assigned to the SA or RE conditions (within a genotype), resulting in the necessity to confirm that training results were similar between both conditions within each genotype (Fig. 3A and 3B). The text previously on line 247 was removed from the manuscript and the following text was included in the manuscript:

“Genotypes were matched on their performance during nose-poke training (stage 2), as we aimed to assess genotype differences during the extinction test only (stage 4). After nose-poke training, all mice were assigned to either the SA or the RE condition (within a genotype). Analyses confirmed that learning was not different between both conditions after this random assignment.” [Line 243- 246]

11. Grooming assessments during competing behaviors. Distinct measurements of grooming time during the performance of other tasks reflects more about behavioral competition than simple compulsive grooming behavior, and when you consider the very small amounts of grooming time exhibited in the SA or RE tests as well as the EPM, the only true grooming difference seems to be in the Open Field with a longer testing time that provides the assessment of grooming periods without behavioral competition. It would be better to present Figure 5 data as % time grooming to highlight this caveat and include the total time of the test in the figure legend.

The reviewer points out that grooming was evaluated within different context and time frames. Similar to the reviewer, we assume that grooming in the open field strongly indicates the baseline grooming levels of the animals. However, we also analyzed grooming throughout the behavioral tests to gain some insight into when, and how much, animals engage in grooming behavior compared to other behaviors (e.g., exploration of the EPM or reward exploitation in the SA task). Moreover, we were interested in this point, because grooming during different trial epochs (presentation of cue light or feedback signal) may have negatively affected task performance. Figure 5 shows that SAPAP3-/- groom more than WT littermates, but suggests that the amount of grooming during the SA task is not related to the SA test outcome. As requested, to improve transparency about time frames, we now include the duration of all tests in the figure legend [Lines: 625; 642-644]

Furthermore, we would like to add that the statistical tests performed are not affected by whether overall grooming duration would be considered low or not, but instead consider the variation of the measured outcome. Thus, we believe that these significant differences are very much useful independent of their absolute size (also see above).

12. Compulsive responding as defined by ENP in UCT. Compulsive responding as defined only by the ENP in UCT seems a bit limiting in scope given that the data in Fig. 4 shows only 13% of trials (50 trials) as uncompleted, thus limiting the interpretation given that Saoap3 KO mice do not show more compulsive responding except in the completed trials for the RE groups.

The reviewer expresses concern about the number of ENP in UCT. However, the SA paradigm is of comparative nature rather than being based on achieving a certain threshold for indicators of compulsivity. Our results are comparable to those of previous studies by others (e.g. Joel 2006; Albelda & Joel 2012) and although based on a subset of total trials, findings of this nature have been consistent for at least 17 independent studies across several different laboratories.

13. Correlations with self-grooming. The restricted range of self-grooming times during the final extinction test limit the strength of the correlational analyses such that they are drastically affected by measures at the extremes. Consider removing as they do not add significant information to the data.

There was no restriction for grooming or responding during the final extinction test (stage 4). Therefore, we wanted to describe all of the observed behavior. Importantly, to answer our research question about the unitary or heterogeneous nature of compulsivity it is necessary to evaluate the expression of different measures of compulsivity in relation to each other.

14. Line 209, the result subheadings “Training C57Bl6 mice” is not informative. Please use subheadings that are specific and informative and that capture the main conclusion of the result section.

We improved the subheading: “C57BL6 mice readily acquired the response requirements in the SA task.” [Line 212]

14-20. Editorial changes.

14. Line 210, change “Fig. 2” to “Fig. 2A”.

We corrected the link to the figure: “Fig. 2A” [Line 213]

15. Line 243, change “Training WT and SAPAP3-/-” to an informative subheading. Please consider to add “mice” to this title and through the manuscript.

We improved the sub-heading: “Attenuation of the feedback signal was similar in SAPAP3-/- and WT.” [Line 242]

16. Line 208 and 242, please remove experiment 1 and 2.

We removed the subheadings: experiment 1 and 2.

17. Figure 2, you may want to use WT-RE and WT-SA labels for Fig. 2. It is a bit confusing that wildtype mice are named “C57Bl6-SA” in Figure 2 and “WT-SA” in other figures.

We made this discrimination to indicate that in a first step we implemented the SA task in a standard laboratory C57BL6 mouse, and in a second step used this newly implemented task to compare our SAPAP3-(- mouse model for compulsivity with their normal WT littermate controls. As for this, we would like to keep this differentiation in the manuscript.

18. Figure 3, consider to add the genotype to the key of the figure panels by changeing “SA” to “WT-WT” or “SAPAP3-/-SA”.

For coherence and simplicity of the figures we would like to provide information about the genotype in the figure title (SAPAP3-/-, WT), using the figure legend to indicate the specific condition (SA, RE).

19. Line 262, change “Fig 3c” to “Fig.3C”.

We corrected the link to the figure: “Fig. 4C” [Line 265]

20. Figure legends, indicate the n number of all genotypes in the figure legends.

We agree with this suggestion and added the n for all animals to the figure legends.

[ Lines: 596; 624-625; 643-644 ]

21. The title seems too long and technical for the general audience. General audience does not know that the Sapap3 ko is a genetic model of obsessive-compulsive disorder. For example, “Evidence of distinct forms of compulsivity in the Sapap3 knockout mouse model of obsessive-compulsive disorder” would be more appealing and easier to understand.

We agree with this suggestion of the reviewer and changed the title to: “Evidence for distinct forms of compulsivity in the SAPAP3 mutant-mouse model for obsessive-compulsive disorder”
